# A clinical assessment of the therapeutic effects of Ashwagandha root extract on cognitive performance, sleep, and fatigue in children aged 6–12 years

**DOI:** 10.3389/fnut.2026.1742138

**Published:** 2026-03-11

**Authors:** Amit Saxena, Adrian Lopresti, Mumtaz Sharif, Neelu Elon, Ravleen Suri, Deepak K. Langade

**Affiliations:** 1 Department of Paediatric Medicine, D. Y. Patil University School of Medicine, Navi Mumbai, Maharashtra, India; 2 Clinical Research Australia, Duncraig, WA, Australia; 3 Department of Pharmacology, D. Y. Patil University School of Medicine, Navi Mumbai, Maharashtra, India

**Keywords:** Ashwagandha, children, cognition, COMPASS, memory, PROMIS, *Withania somnifera*

## Abstract

**Introduction:**

Ashwagandha (*Withania somnifera L.Dunal*) is an adaptogenic herb known to reduce stress and enhance well-being in adults.

**Methods:**

This randomized, double-blind, placebo controlled, parallel-group trial evaluated the efficacy and safety of standardized Ashwagandha root extract (ARE) in children with parent-reported concerns related to attention, concentration, or memory. Eight-five healthy children aged 6-12 years were randomized to receive ARE gummies (*n* = 42; 150 mg twice daily) or identical placebo gummies (*n* = 43) for 8 weeks. Primary outcomes included attention, memory, and executive function assessed using the Computerized Mental Performance Assessment System (COMPASS). Secondary outcomes included overall functioning and well-being assessed using the Strengths and Difficulties Questionnaire (SDQ), Behavior Rating Inventory of Executive Function, Second Edition (BRIEF2 Parent version), Sleep Disturbance Scale for Children (SDSC), and Patient-Reported Outcomes Measurement Information System - Fatigue Scale. Safety was evaluated based on self-reported adverse events.

**Results:**

Among 73 participants who completed the study (ARE, *n* = 39; placebo, *n* = 34), ARE supplementation significantly improved speed of information processing (*p* = 0.040). Improvements were also observed in delayed word recall (*p* = 0.038, *d* = 0.59), Stroop task accuracy (*p* = 0.021, *d* = 0.61), Corsi block span (*p* = 0.013, *d* = 0.66), and choice reaction time accuracy (*p* = 0.005, *d* = 0.75). Additionally, SDSC scores improved, indicating better parent-reported sleep quality (*p* = 0.035). No significant adverse events were reported.

**Conclusion:**

These findings suggest that an eight-week supplementation with ARE is well tolerated and may enhance cognitive performance and sleep quality in children.

**Clinical trial registration:**

The trial was prospectively registered with the Clinical Trials Registry of India (CTRI/2021/10/037126; dated 06/10/2021; CTRI) and the Australian and New Zealand Clinical Trials Registry (Reg. No.: ACTRN12621000656831; ANZCTR–Registration).

## 1 Introduction

Cognition refers to the state and process involved in acquiring and understanding knowledge. It includes conscious and unconscious activities like thinking, learning, remembering, abstraction, judgment, problem-solving, language, imagination, perception, planning, and execution (1). All of these activities usually develop in early childhood and continuously progress through social, educational and environmental interactions (2).

Approximately 15% of children experience poor working memory, which often manifests as inattentiveness, distractibility, and difficulty completing tasks that require sustained focus. Despite these challenges, such children typically exhibit normal emotional regulation, self-esteem, and social integration, though they may appear withdrawn in large groups. More than 80% struggle with reading and mathematics, often making limited academic progress without meeting criteria for special educational support (3). In addition to learning difficulties, they may exhibit broader cognitive deficits, including low IQ and impairments in executive functions such as planning, problem solving, and attention regulation. While causality remains unclear, limited working memory capacity may underlie these widespread challenges (4).

The World Health Organization reports that behavior-related issues are common in children (4.4% in 10 to 14-year-olds and 5.5% in 15 to 19-year-olds), which can impair attention, memory, and decision-making, leading to cognitive disruption (5). Early identification of such disruptions is essential. An early cognitive assessment plays a key role in identifying difficulties and reducing the long-term impact (6). Following cognitive assessment, it is important to carefully select treatment modalities that will be devoid of adverse effects, as interventions-related adverse events introduced during early life can have a lasting impact across an individual’s lifespan (7).

The current pharmacological treatment for managing attentional difficulties in children includes stimulant medications such as dexamphetamine and managing emotional and behavioral disturbances, such as antidepressant and stimulant medications, are associated with a range of adverse effects, such as sleep disturbances, appetite suppression, gastrointestinal upset, emotional blunting, and headaches (8–10). As a result, there is growing interest in exploring herbal or plant-based formulations that offer therapeutic benefits with a potential lower toxicity risk with long-term use. Herbal adaptogens such as *Withania somnifera* (Ashwagandha) have demonstrated promising effects in improving cognitive function (11), reducing stress (12) and improving mood (13) in adults; however, research in pediatric populations is limited (14). While most existing medications to improve cognition have side effects, complementary and alternative options such as Ashwagandha are gaining popularity as effective adaptogens. Biochemically, Ashwagandha is classified as a phytochemical with potential nootropic effects (15). Ashwagandha (*Withania somnifera L.Dunal*) is a popular Ayurvedic Rasayana and belongs to a subgroup of Rasayanas known as Medhya Rasayanas. The term “Medhya” refers to the mind and its capacities for cognition. Ashwagandha is known to enhance cognitive abilities and memory (16). The cognitive-enhancing impact of MedhyaRasayanas is most evident in children with memory impairments, as well as in individuals experiencing memory deterioration due to head trauma, chronic sickness, or old age (17).

The actions of Ashwagandha root extract (ARE) are attributed to a range of biologically potent compounds, such as Withanolides, alkaloids, and flavonoids (17–19). These compounds have the ability to alter the activity of gamma-aminobutyric acid (GABA), the hypothalamic–pituitary–adrenal (HPA) axis, the neuroendocrine system, and oxidative stress pathways (20–23). According to Kale *et al*. (2024), ARE was found to be both safe and effective in improving cognition, energy levels, and mood in adults, based on a prospective, randomized, placebo-controlled study (11). Despite the proven therapeutic benefits in adults, the studies on the effect of ARE in children are limited. To address this gap, a placebo-controlled trial using computer-based tasks and parent-reported questionnaires was conducted to investigate the safety and possible cognitive and sleep-promoting effects of an 8-week intake of an ARE dosage in children aged 6 to 12. The primary objective of this study was to assess the efficacy of ARE on cognitive performance in children, while the secondary objective was to evaluate its safety and tolerability. Importantly, the present study focuses on children with subclinical, parent-reported attentional and memory concerns rather than formally diagnosed neurodevelopmental disorders.

## 2 Materials and methods

### 2.1 Study design, settings, and procedures

This study was an 8-week, parallel-group, randomized, double-blind, placebo-controlled, prospective trial conducted at two sites, the Department of Pharmacology of D. Y. Patil Medical College and Hospital in Navi Mumbai, India, and Clinical Research Australia in Perth, Western Australia. The trial protocol received approval from both the Human Research Ethics Committee at the National Institute of Integrative Medicine (approval number: 0079E_2020) and the Institutional Ethics Committee of D. Y. Patil Medical College, Navi Mumbai (IEC Ref. No. DYP/IEC/23/2021). Informed written consent was obtained from all participants and their parents or legal guardians before commencement of the study. The study was conducted in compliance with the guidelines established by the Declaration of Helsinki and Good Clinical Practice (GCP) and was prospectively registered with the Clinical Trials Registry of India (CTRI/2021/10/037126; dated 06/10/2021; CTRI) and the Australian and New Zealand Clinical Trials Registry (Reg. No.: ACTRN12621000656831; ANZCTR - Registration).

**Figure 1:**
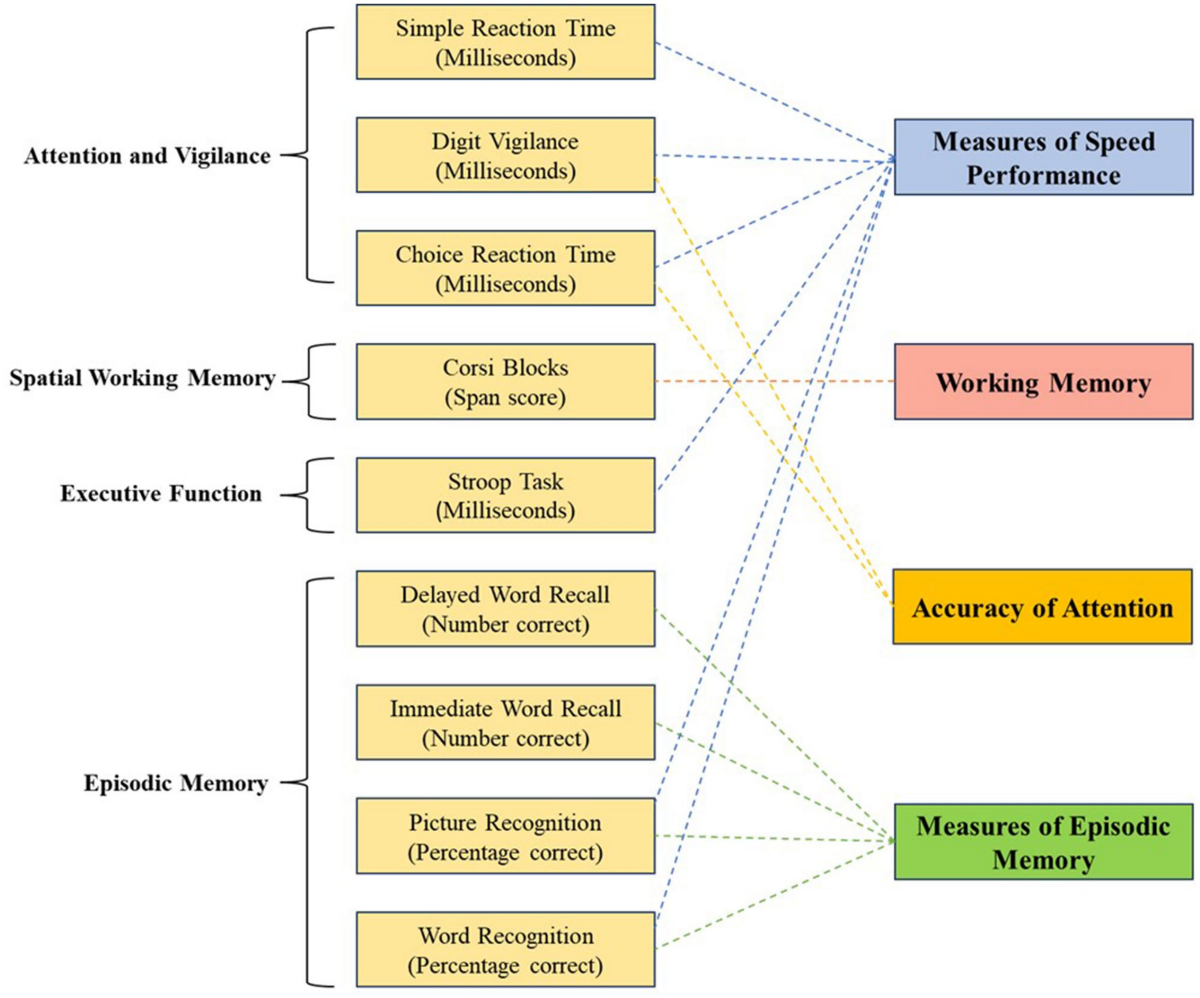
COMPASS categorizations and scores. The figure illustrates the cognitive tasks used to evaluate attention and vigilance, spatial working memory, executive function, and episodic memory. Individual task outcomes (reaction time, span scores, and accuracy measures) are grouped to derive composite measures of speed performance, working memory, accuracy of attention, and episodic memory, providing an integrated assessment of cognitive performance across domains.

Participants and their parents or guardians attended assessment visits at baseline, week 4, and week 8. All parameters were assessed at each visit, except for the Computerized Mental Performance Assessment System (COMPASS) task, which was measured only at baseline and week 8. All visits were planned between 8:00 a.m. and 4:00 p.m., and to ensure consistency with pre- and post-assessments were completed at similar times of day. The participants were instructed to consume food 3 h prior to each visit and to maintain a similar diet for all time points of assessments. At each visit, the weight, height, and blood pressure of the participants were measured, followed by cognitive tasks using the COMPASS as detailed in Figure 1, and the Sleep Disturbance Scale for Children (SDSC). Parents or guardians completed the Strengths and Difficulties Questionnaire, Parent Version (SDQ), Behavior Rating Inventory of Executive Function, 2nd Ed (BRIEF2), and PROMIS Fatigue Scale relating to their child’s behavior and symptoms. To ensure consistency, the same parent completed questionnaires at all-time points.

### 2.2 Inclusion criteria

Healthy male and female children aged 6 to 12 years with a body mass index (BMI) between 18 to 30 kg/m^2^ were selected for the study. Children attended mainstream schooling, but had parent-reported concerns about their attention, concentration or memory. There was no plan to begin any new treatment during the study period, and both the parent or guardian and child were fluent in English. In addition, both the parent or guardian and the participant were required to provide written informed consent.

### 2.3 Exclusion criteria

Children with a history of chronic and clinically significant medical illnesses, such as diabetes, organic brain disorders, cardiovascular diseases, or seizures, or those using pharmaceutical drugs expected to affect behavior and learning were excluded. Additionally, those diagnosed with a psychiatric disorder (including attention deficit hyperactivity disorder) within the past 12 months or a diagnosis of any learning disorder were excluded. Moreover, children undergoing significant familial or societal pressures (e.g., recent parental separation or a significant illness in the family), who had undergone any surgical procedure within the previous year, and who were taking nutraceutical supplements were excluded.

### 2.4 Study participants

Participants were recruited through social media advertisements, email databases, and from the outpatient department at D. Y. Patil Medical College, where parents had reported concerns about their child’s attention, concentration, and memory. Comprehensive explanations regarding the study’s aims, risks, benefits, and anticipated results were provided to all participants and their parents or guardians before enrolment. A total of 85 healthy children (48 males and 37 females) were enrolled and randomized to receive either an ARE (*n* = 42) or a placebo (*n* = 43).

### 2.5 Interventions

The intervention, consisting of a standardized ARE, KSM-66 Ashwagandha®, was supplied by Ixoreal Biomed Inc. KSM-66 is the commercially available highest concentration root-only extract of Ashwagandha, and is produced by a green chemistry method (aqueous-based extraction process) that is devoid of alcohol or chemical solvents. The product contains an optimum amount of Withanolides (>5%) precisely estimated by the High-Performance Liquid Chromatography method. The product is a light yellowish powder with a neutral taste, which makes it suitable for children’s administration in the form of gummies. The drug-to-extract ratio is 12:1. Both the ARE and placebo gummies were identical in appearance, matched for color, shape, and size. Each gummy of the active intervention contained 150 mg of standardized aqueous extract from the roots of Ashwagandha, while the placebo consisted of pectin and honey. The participants were informed to take one gummy twice daily with breakfast and dinner. The protocol compliance was monitored through a mobile phone application, along with the return and count method. In addition, compliance was verified by the return-and-count method of unused gummies at each study visit.

### 2.6 Sample size calculations

This study was designed as an exploratory, hypothesis-generating trial; therefore, no formal *a priori* sample size calculation was performed (24). Based on feasibility considerations across two sites, a planned enrolment target of approximately 80 participants (40 per group) was set to evaluate tolerability and obtain preliminary estimates of efficacy. Due to higher-than-anticipated recruitment rates, a total of 85 participants were ultimately randomized (ARE, *n* = 42; placebo, *n* = 43).

### 2.7 Randomization and blinding

Participants and researchers were blinded throughout the study. A 1:1 randomization was independently performed for each trial site using computer-based randomization software (Rando 1.2; R. Raveendran, 2004) (25), with stratification by site. Both the investigational product and placebo were visually identical and packaged in indistinguishable, coded containers labeled only with the participant’s serial number (study ID). Upon enrolment, participants received the corresponding coded pack. The active product and matching placebo were manufactured and packaged by Ixoreal Biomed Inc. to ensure uniformity and maintain blinding integrity across sites. Randomization codes were generated by an independent statistician and remained concealed until data analysis. The allocation sequence was implemented by site personnel not involved in outcome assessments, ensuring that investigators, participants, and study staff remained blinded throughout the study. Randomization codes were generated by an independent statistician and remained concealed until data analysis. Emergency unblinding procedures were in place but were not required during the study.

### 2.8 Outcome measures

#### 2.8.1 Primary outcome measure

##### 2.8.1.1 Computerized mental performance assessment system

The COMPASS (Northumbria University, Newcastle upon Tyne, United Kingdom, Software Version 2.0) is a software application that integrates a battery of tests to assess working memory, performance speed, attention, and visual learning (22, 23, 26). At baseline and week 8, participants underwent a short practice session to familiarize themselves with the tasks and then completed the full COMPASS evaluation. The cognitive tasks and their corresponding scores are detailed in Figure 1.

#### 2.8.2 Secondary outcome measure

##### 2.8.2.1 Strengths and difficulties questionnaire parent version

The SDQ is a concise emotional and behavioral assessment tool consisting of 25 items, used to evaluate children and adolescents between the ages of 4 and 17 years (27). It comprises five sub-scales, each scored from 0 to 10, that evaluate various aspects of mental health concerns like emotional symptoms, conduct problems, hyperactivity/inattention, peer relationship problems, and prosocial behavior. The SDQ has been widely utilized as a screening instrument and to monitor treatment outcomes, frequently employed worldwide for therapeutic and research purposes. For this study, the parent version of the scale was used and was completed at baseline, week 4, and week 8 (27, 28).

##### 2.8.2.2 Sleep disturbance scale for children

The SDSC is a 26-item parent-rated inventory scored on a 5-point Likert scale with scoring points as Never, Occasionally, Sometimes, Often, and Always (29). It contains six subdomain scores: sleep breathing disorders (SBD), disorders of excessive somnolence (DOES), difficulty in initiating and maintaining sleep (DIMS), sleep–wake transition disorders (SWTD), disorders of arousal (DoA), and sleep hyperhidrosis (SH). These subscale scores are summed to generate a ‘total sleep difficulties’ score (29, 30). The SDSC was completed on the child at baseline, week 4, and week 8. The higher score indicates greater severity or frequency of sleep disturbances.

##### 2.8.2.3 Patient-reported outcomes measurement information system–fatigue scale

The PROMIS-Fatigue is a 23-item questionnaire rated by parents (on a 0–10 numeric rating scale) concerning their child’s fatigue and energy levels related to daily activities (31). This validated questionnaire assesses the child’s fatigue over the last 7 days using a 5-point Likert scale (31, 32). Higher scores indicate greater fatigue severity. The PROMIS-Fatigue was completed by the parent or guardian at baseline, week 4, and week 8.

##### 2.8.2.4 Behavior rating inventory of executive function 2nd ed (parent version)

The BRIEF-2 is a validated questionnaire consisting of sixty-three items that assesses the impairment of executive function in children and adolescents aged 5–18 years. The BRIEF-2 calculates scores for behavior regulation (inhibition, self-monitoring), emotional regulation (shift, emotional control), and cognitive regulation (initiation, working memory, planning/organization, task monitoring, and organization of materials) (33). The BRIEF-2 was completed by the parent or guardian at baseline and week 8.

##### 2.8.2.5 Adverse events

Participants and parents were instructed to promptly report any adverse events that occurred throughout the entire study duration, including the follow-up period. Clinical safety was evaluated by assessing any adverse events reported or observed during this period.

### 2.9 Statistical analysis

Data analysis on the per-protocol (PP) datasets was conducted using MedCalc Statistical Software (version 18.1) (MedCalc Software bvba, Ostend, Belgium, 2018),^1^ a Windows-based statistical program. Efficacy analyses were conducted using a per-protocol dataset comprising participants who completed baseline and week-8 assessments. No intention-to-treat analysis was performed, as missing post-baseline cognitive data could not be reliably imputed due to the nature of computerized task outcomes.

Measured data were presented as means with one standard error (SE). Demographic data and scores for various scales were analyzed for differences using the independent sample t-test for both datasets. Pairwise comparisons of baseline versus post-treatment values were performed using the paired t-test. Cohen’s d was calculated to estimate the effect size between the study and control groups, quantifying the treatment effect. Multivariate analyses were conducted to assess the overall effect of treatments on cognitive function. Given the exploratory nature of the study and the multiple cognitive endpoints assessed, no formal adjustment for multiple comparisons was applied. Findings should therefore be interpreted cautiously, with an emphasis on effect size magnitude and consistency rather than isolated *p*-values.

## 3 Results

**Figure 2:**
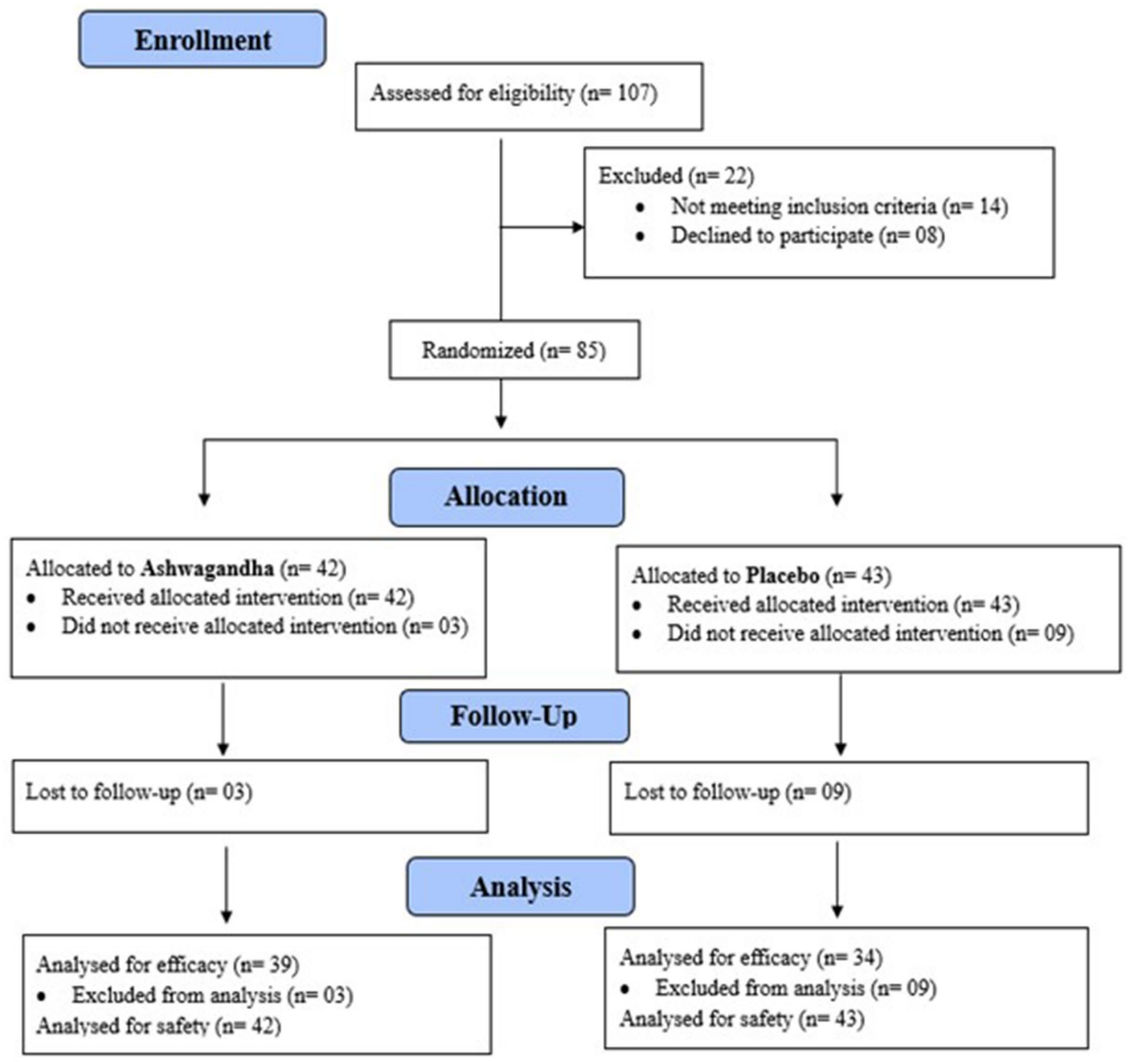
CONSORT (2010) patient flow diagram. The diagram summarizes participant enrolment, randomization, follow-up, and analysis. Of 107 children assessed for eligibility, 85 were randomized to receive Ashwagandha root extract (*n* = 42) or placebo (*n* = 43). Losses to follow-up resulted in 39 and 34 participants included in the per-protocol efficacy analysis for the Ashwagandha root extract and placebo groups, respectively, while all randomized participants were included in the safety analysis.

Details of participant recruitment, treatment allocation, and treatment discontinuation are presented in the CONSORT chart Figure 2. From India (*n* = 44), 22 were allocated to each of the two groups, whereas from Australia (*n* = 41), 20 were allocated to ARE and 21 to the placebo group. In the ARE group, one participant experienced mild skin-related symptoms, two disliked the taste of gummies, and seven missed the final follow-up without providing a reason. In the placebo group, one participant reported stomach discomfort, another reported fatigue, and two missed the final appointment with no clear explanation.

### 3.1 Baseline scores in randomized participants

The two groups were comparable with respect to the patient demography and the baseline scores for different parameters (Table 1).

**Table 1:** Patient characteristics and baseline scores in randomized participants (*n* = 85).

Baseline parameters	Ashwagandha root extract (*n* = 42)		Placebo (*n* = 43)		*t*-test
Gender	*n* (%)		*n* (%)		–
Male	26 (61.90%)		22 (51.20%)		
Female	16 (38.10%)		21 (48.80%)		
	Mean	SE	Mean	SE	*p*
Age (yrs.)	8.90	0.29	8.37	0.31	0.212
SBP (mm Hg)	102.07	1.68	99.63	1.52	0.284
DBP (mm Hg)	63.17	1.37	63.90	2.04	0.766
BMI (kg/m^2^)	18.46	0.44	19.09	0.46	0.326
COMPASS					
Episodic memory					
Word immediate recall (%)	45.37	2.16	44.32	3.65	0.802
Word delayed recall (%)	25.37	2.10	26.33	2.86	0.781
Word recognition (%)	80.12	1.87	80.24	2.53	0.969
Picture recognition (%)	92.07	0.92	91.63	1.70	0.816
Executive function					
Stroop task (%)	92.94	1.67	92.20	2.44	0.803
Working memory					
Corsi blocks span score	4.53	0.19	4.31	0.19	0.412
Speed of processing information					
Simple reaction time (ms)	824.50	128.43	811.28	98.69	0.935
Choice reaction time (ms)	1043.93	96.33	1093.01	83.40	0.702
Picture recognition (ms)	1701.56	96.62	1714.69	129.31	0.935
Word recognition (ms)	2256.42	180.31	2279.15	243.32	0.940
Digit vigilance (ms)	541.84	8.27	542.31	11.15	0.973
Stroop task (ms)	1339.38	74.36	1315.21	57.97	0.799
Accuracy of attention					
Choice reaction time (%)	91.46	2.28	91.33	1.59	0.965
Digit vigilance (%)	36.05	2.94	35.26	3.52	0.864
Patient-reported outcomes					
SDQ score	16.14	0.68	16.05	0.57	0.913
SDSC score	40.50	1.94	40.26	1.57	0.922
PROMIS score	37.21	1.96	37.79	2.37	0.852
BRIEF-2 (Parent)					
BRI score	22.07	0.71	22.23	0.95	0.893
ERI score	29.62	0.81	29.93	1.17	0.828
CRI score	62.45	1.76	62.44	2.35	0.997
GEC score	114.14	2.54	114.60	4.12	0.925

BMI, Body mass index; BRIEF-B, Behavior rating inventory of executive function (Children); BRI, Behavioral regulation index; COMPASS, Computerized mental performance assessment system; DBP, Diastolic Blood Pressure; ERI, Emotion Regulation Index; CRI, Cognitive regulation Index; GEC, Global executive composition status; PROMIS, Patient-Reported Outcomes Measurement Information System; SBP, Systolic Blood Pressure; SDQ, Strengths and Difficulties Questionnaire; SDSC, Sleep Disturbance Scale for Children.

### 3.2 Change in COMPASS scores

Table 2 presents the scores for different COMPASS tasks at baseline and week 8. A multivariate analysis revealed significantly greater improvements in the ARE group compared to the placebo group in the Speed of Processing Information (*p* = 0.040). This was demonstrated by greater improvements in choice reaction time between the groups (*p* = 0.040, d = 0.54), and strong trends of improvement between the groups in response times on picture recognition (*d* = 0.44), word recognition (*d* = 0.40), and digit vigilance (*d* = 0.41). However, multivariate analyses did not demonstrate group differences in changes in the accuracy of attention (*p* = 0.784) and episodic memory (*p* = 0.206). An analysis of performance on different cognitive tasks revealed about participants in the ARE group experienced significantly greater improvements in the percentage of recalled items on the word delayed recall task (*p* = 0.038, *d* = 0.59), the percentage of correct responses on the Stroop task (*p* = 0.021, *d* = 0.61), the span score on the Corsi Blocks task (*p* = 0.013, *d* = 0.66), and the percentage of correct responses for choice reaction time (*p* = 0.005, *d* = 0.75).

**Table 2:** Compass scores in two groups in PP dataset (*n* = 73).

Parameters	Ashwagandha root extract (*n* = 39)					Placebo (*n* = 34)				Effect size	Between group	Multivariate
	Mean	SE	Change	SE	*p* ^a^	Mean	SE	Change	*p* ^a^		*p* ^b^	*p* ^b^
Episodic memory												
Word immediate recall (%)												
Baseline	45.00	2.19	–	–	–	42.90	4.10	–	–	0.115	0.637	0.206
8 weeks	49.72	2.39	−3.89	2.65	0.151	45.28	4.26	−5.68	0.096	0.254	0.333	
Word delayed recall (%)												
Baseline	24.21	2.12	–	–	–	25.58	3.23	–	–	−0.094	0.713	
8 weeks	39.28	2.40	−14.83	2.73	<0.0001	31.50	2.29	−9.75	0.007	0.594	0.038	
Word recognition (%)												
Baseline	79.87	1.99	–	–	–	80.78	2.92	–	–	−0.064	0.792	
8 weeks	88.06	1.40	−6.94	2.28	0.004	82.50	2.80	0.00	1.000	0.494	0.059	
Picture recognition (%)												
Baseline	91.97	0.97	–	–	–	90.94	2.04	–	–	0.116	0.631	
8 weeks	95.28	1.70	−3.06	1.95	0.127	92.69	1.97	0.58	0.819	0.255	0.325	
Executive function												
Stroop task (%)												
Baseline	93.22	1.77	–	–	–	91.02	2.99	–	–	0.158	0.513	–
8 weeks	97.57	0.60	−4.58	1.79	0.015	93.56	1.82	−3.65	0.290	0.608	0.021	
Working memory												
Corsi blocks span score												
Baseline	4.53	0.20	–	–	–	4.37	0.18	–	–	0.142	0.557	-
8 weeks	5.00	0.17	−0.39	0.20	0.059	4.36	0.18	0.17	0.393	0.655	0.013	
Speed of processing information												
Simple reaction time (ms)												
Baseline	830.62	138.18	–	–	–	696.79	65.94	–	–	0.198	0.413	0.040
8 weeks	628.14	69.57	208.79	142.57	0.152	694.55	60.84	−12.53	0.817	−0.176	0.496	
Choice reaction time (ms)												
Baseline	1061.71	101.77	–	–	–	1089.16	91.14	–	–	−0.047	0.844	
8 weeks	812.93	34.35	235.76	91.23	0.014	961.88	68.78	78.60	0.456	−0.540	0.040	
Picture recognition (ms)												
Baseline	1713.42	103.45	–	–	–	1827.96	152.47	–	–	−0.153	0.526	
8 weeks	1398.51	75.70	311.39	120.12	0.014	1625.95	117.33	117.43	0.588	−0.438	0.094	
Word recognition (ms)												
Baseline	2328.22	189.66	–	–	–	2344.88	286.82	–	–	−0.012	0.960	
8 weeks	1564.76	98.64	690.61	182.24	0.001	1834.43	149.39	160.43	0.306	−0.404	0.122	
Digit vigilance (ms)												
Baseline	540.65	8.64	–	–	–	541.40	14.13	–	–	–	0.963	
8 weeks	475.20	21.93	68.64	20.61	0.002	523.78	18.63	30.34	0.086	−0.413	0.118	
Stroop task (ms)												
Baseline	1313.99	75.71	–	–	–	1342.75	70.33	–	–	–	0.784	
8 weeks	1219.79	59.95	111.23	87.64	0.213	1293.74	76.16	−28.54	0.736	−0.199	0.443	
Accuracy of attention												
Choice reaction time (%)												
Baseline	91.89	2.44	–	–	–	90.31	1.89	–	–	–	0.622	0.784
8 weeks	96.67	0.76	−5.14	2.73	0.068	92.44	1.36	−1.28	0.544	0.747	0.005	
Digit vigilance (%)												
Baseline	34.98	2.99	–	–	–	31.95	3.89	–	–	–	0.534	–
8 weeks	48.76	8.96	−13.51	9.64	0.170	38.10	8.27	−3.16	0.709	0.216	0.404	

Significant covariate effects were observed in the change in Visit 3 for: Word Recall Delayed (*p* = 0.018), Digit Vigilance RT (*p* = 0.031). ^a^Within group pairwise comparison; ^b^Between group comparisons for change from baseline; COMPASS: Computerized mental performance assessment system.

### 3.3 Change in SDQ total score

As demonstrated in Table 3, there was no statistically significant between-group difference in the change in the SDQ score from baseline to week 8 (*p* = 0.075). However, in the ARE group, there was a statistically significant reduction in the SDQ score from baseline to week 8 (*p* = 0.015), but no statistically significant change in the placebo group (*p* = 0.392).

**Table 3:** SDQ, SDSC, PROMIS and BRIEF-2 scores in two groups in PP dataset (*n* = 73).

Parameters	Ashwagandha root extract (*n* = 39)					Placebo (*n* = 34)				Effect size	Between group	Multivariate
	Mean	SE	Change	SE	*p* ^a^	Mean	SE	Change	*p* ^a^		*p* ^b^	*p* ^b^
SDQ												
Baseline	16.05	0.72	–	–	–	16.24	0.68	–	–	–	0.855	0.766
4 weeks	14.68	0.67	1.37	0.87	0.129	15.89	1.10	−0.667	0.605	−0.252	0.338	
8 weeks	13.15	0.67	2.63	1.04	0.015	15.24	0.98	1.143	0.392	−0.428	0.075	
SDSC												
Baseline	40.56	2.03	–	–	–	40.50	1.79	–	–	–	0.981	0.567
4 weeks	37.26	1.59	3.42	2.41	0.164	39.62	1.66	2.379	0.157	−0.241	0.308	
8 weeks	34.67	0.95	6.08	2.01	0.004	38.24	1.40	4.103	0.012	−0.506	0.035	
PROMIS												
Baseline	37.41	2.06	–	–	–	37.06	2.63	–	–	–	0.916	0.227
4 weeks	29.90	1.27	7.71	1.52	<0.0001	34.27	2.05	3.750	0.070	−0.443	0.065	
8 weeks	28.85	1.25	8.79	1.74	<0.0001	32.94	1.92	5.321	0.029	−0.435	0.070	
BRIEF-2												
BRI												
Baseline	21.85	0.66	–	–	–	23.12	1.07	–	–	–	0.302	<0.001
4 weeks	20.32	0.72	1.38	0.46	0.005	21.50	1.21	2.286	<0.0001	−0.220	0.383	
8 weeks	19.45	0.69	2.39	0.50	<0.0001	21.21	1.42	2.571	0.011	−0.302	0.229	
ERI												
Baseline	29.90	0.83	–	–	–	30.85	1.32	–	–	–	0.532	0.266
4 weeks	27.16	0.84	2.95	0.75	<0.0001	28.93	1.53	2.679	0.005	−0.269	0.287	
8 weeks	25.97	0.94	3.92	0.82	<0.0001	28.50	1.65	3.107	0.007	−0.352	0.162	
CRI												
Baseline	62.38	1.87	–	–	–	63.06	2.45	–	–	–	0.825	0.010
4 weeks	57.41	2.23	4.86	1.37	<0.0001	60.89	2.76	3.679	0.047	−0.249	0.325	
8 weeks	54.37	1.89	8.29	1.49	<0.0001	59.71	3.18	4.857	0.016	−0.381	0.131	
GEC												
Baseline	114.13	2.57	–	–	–	117.03	4.55	–	–	–	0.568	0.018
4 weeks	104.89	3.13	9.19	2.10	<0.0001	111.32	5.01	8.643	0.003	−0.285	0.259	
8 weeks	99.79	2.72	14.61	2.29	<0.0001	109.43	5.60	10.536	0.001	−0.417	0.099	

Significant covariate effects were observed in the change at Visit 2 and Visit 3 for: BRI Visit 2 (*p* < 0.001), BRI Visit 3 (*p* = 0.013), CRI Visit 2 (*p* = 0.003), and GEC Visit 2 (*p* = 0.016). ^a^Within group pairwise comparison; ^b^Between group comparisons for change from baseline. BRIEF-B, Behavior rating inventory of executive function (Children); BRI, Behavioral regulation index; ERI, Emotion Regulation Index; CRI, Cognitive Regulation Index; GEC, Global executive composition status; SDQ, Strengths and Difficulties Questionnaire; SDSC, Sleep Disturbance Scale for Children.

### 3.4 Change in SDSC total score

As demonstrated in Table 3, there was a statistically significant between-group difference in the change in the SDSC score from baseline to week 8 (*p* = 0.035, *d* = 0.51). In the ARE group, the SDSC score reduced from baseline to week 8 (*p* = 0.004), and in the placebo group, there was a statistically significant but smaller 10.1% reduction (*p* = 0.012).

### 3.5 Change in PROMIS-fatigue score

As demonstrated in Table 3, there was no statistically significant between-group difference in the change in the PROMIS-fatigue score from baseline to week 8 (*p* = 0.070). However, in the ARE group, there was a statistically significant reduction in the PROMIS-fatigue score from baseline to week 8 (*p* < 0.0001), and in the placebo group, there was a statistically significant but smaller reduction (*p* = 0.029).

### 3.6 Change in BRIEF-2 scores

As demonstrated in Table 3, there was no statistically significant between-group difference in the change in any BRIEF-2 score from baseline to week 8. However, in the ARE group, there were statistically significant within-group changes from baseline to week 8 on all BRIEF-2 scores (*p* < 0.0001), but no statistically significant within-group changes in the placebo group, except for Emotion Regulation Index (*p* = 0.007) and Global executive composition status (*p* = 0.001).

### 3.7 Safety outcomes, adverse events, and treatment discontinuation

During the study, ARE and the placebo were well-accepted and tolerated. No serious adverse events/episodes were reported in the study period and during the follow-up. However, three participants in the ARE group experienced mild stomach pain, 1 complained of tiredness and breathing problems, and 1 reported tonsillitis. In the placebo group, one participant each reported rashes, abdominal pain, and ear pain. All reported adverse events were mild and transient. Events such as tiredness and breathing discomfort were assessed by investigators and were not considered related to the study product. No participant discontinued due to product-related adverse effects. Overall, the frequency and nature of adverse events were comparable between the ARE and placebo groups, with no safety signals or treatment-related concerns identified during the 8-week intervention period.

## 4 Discussion

The current randomized, double-blind, placebo-controlled clinical study provides evidence of the efficacy and safety of ARE (150 mg twice daily for 8 weeks) in improving cognitive performance and sleep quality in healthy children aged 6–12 years with parent-reported attention and memory concerns. The 150 mg dose of standardized Ashwagandha (*Withania somnifera L.Dunal*) root extract was selected based on pharmacological and clinical considerations. The extract used in this study is standardized to withanolides, the primary bioactive constituents, allowing biological activity at relatively low doses compared with non-standardized preparations. Previous human clinical studies evaluating stress, sleep, and cognitive outcomes have demonstrated efficacy and good tolerability at daily doses in the low-to-moderate range of standardized AREs. For this pediatric, exploratory study, a conservative dose of 150 mg administered twice daily was chosen to balance potential efficacy with safety and tolerability. Ashwagandha (*Withania somnifera*) root extract showed statistical improvements in overall processing speed, and subtests including delayed word recall (a measure of verbal memory), Stroop task (a measure of executive function), and Corsi Block Span (a measure of visual working memory).

This was the first study examining the efficacy of ARE as a stand-alone intervention on sleep and cognition in children. Positive effects of ARE supplementation on cognitive performance (11, 34, 35) and sleep quality (36) have been identified in adults, but its safety and efficacy in children are under-investigated. This has specific relevance as there is increasing interest in the use of natural ingredients for children, but unfortunately, a lack of robust evidence in the area. Apart from the use of stimulant medications for children with ADHD, there are limited options for children experiencing mild attentional and cognitive difficulties. Although more research is required, the results from this study provide preliminary evidence of safety and efficacy in children. The mechanisms by which Ashwagandha supports cognitive function require further investigation; however, several potential pathways have been proposed, including modulation of neurotransmitter activity and signaling, reduction of stress-related hormones, enhancement of neuroplasticity, and neuroprotective effects (14, 37–39). ARE contains approximately 50 active compounds, including Withanolides (such as steroidal alkaloids and lactones) and sitoindoside, which have been reported to demonstrate memory-related properties by reducing oxidative stress, improving GABAergic signaling, and altering the HPA axis (40). The current study findings were consistent with previous studies reporting significant improvements in cognitive function following ARE supplementation. Choudhary *et al*., (2017) found that ARE at a dose of 300 mg twice daily improved general and immediate memory in adults with mild cognitive impairment, along with enhancing executive function, attention, and information processing speed (13, 41).

Along with cognitive benefits, ARE showed significant improvements in sleep behavior, which was measured using the SDSC (a parent report of their child’s sleep). This is consistent with previous studies demonstrating Ashwagandha’s potential as a natural sleep aid in adults, which are believed to be associated with its GABA-mimetic activity and ability to modulate HPA axis activity (42, 43). The improved sleep quality is of high importance, due to the relationship between sleep and cognitive functioning in children (1). PROMIS-Fatigue scores did not show a statistically significant between-group difference, but within-group analysis showed a reduction in perceived fatigue among ARE-treated children, which could be indirectly linked to improved sleep efficiency.

Significant between-group differences were not seen in the BRIEF-2 domains, whereas the constant within-group improvements in the ARE group suggest an improving trend in executive functioning, mainly in behavioral and cognitive regulation. These effects may reflect that neuroadaptive activity of ARE was not fully captured in an 8-week duration or may require a longer-term evaluation method. Remenapp et al. conducted a placebo-controlled study in adults with self-reported stress, revealing improvements in cortisol levels, cognitive ability, anxiety, depression, self-reported stress, and food cravings following 30-day supplementation with Ashwagandha (44). Additionally, supplementation with ARE (1,000 mg/day for 2 weeks) improved cognitive and psychomotor performance in healthy young males (13, 45). This suggests important mood-stabilizing or anxiolytic effects of ARE in children.

Katz et al. (46) reported significant improvements in attention, impulse control, and cognitive symptoms among children with ADHD following supplementation with a combination of herbal medications, including *Withania somnifera* (12, 46, 47). The pharmacologic interventions show side effects like insomnia, appetite suppression, and irritability (8). ARE offered a well-tolerated, non-pharmaceutical alternative. Importantly, none of the children in the current study reported serious adverse events, showing its safety profile for children.

The present study on the use of non-pharmacological/herbal supplementation for enhancing cognition, stress management, and overall well-being in children was of significant contemporary value. This is particularly important given the concerns surrounding possible adverse reactions, limited approved drugs, and insufficient studies on drug consumption among children and adolescents. Improvements observed in both treatment arms, particularly for parent-reported sleep and fatigue outcomes, likely reflect a combination of expectancy effects, repeated exposure to assessments, parental perception bias, and natural developmental progression over the eight-week study period. This is particularly relevant in children aged 6–12 years, a developmental stage characterized by rapid cognitive maturation. Although randomization mitigates systematic bias, future studies may benefit from age-stratified analyses or narrower age bands to further account for developmental variability.

The findings of this study should be interpreted as preliminary and exploratory rather than definitive evidence of therapeutic efficacy. The study was limited by a relatively small sample size, short intervention duration, and the absence of a formal sample size calculation, which restricts generalizability to the broader population. The reliance on parent- or guardian-reported outcome measures introduces the potential for rater bias, while the analysis of multiple cognitive endpoints increases the risk of Type-I error despite observed consistency across related domains. Additionally, the requirement for English language fluency may have resulted in a more educated and less diverse participant group, further limiting external validity. Attrition and the use of a per-protocol analytical approach may also have influenced effect estimates. Future studies should employ larger, adequately powered designs with intention-to-treat analyses, predefined handling of missing data, and appropriate multiplicity adjustments to confirm these findings and evaluate the long-term safety and efficacy of ARE supplementation.

## 5 Conclusion

The study states that ARE consumption in gummy form for 8 weeks has demonstrated promising outcomes in enhancing memory, cognition, and sleep quality in healthy children. The findings suggest that ARE was well-tolerated and could serve as a safe, non-pharmaceutical herbal intervention to support cognitive performance and overall well-being in this population. With further research and larger clinical trials, ARE holds potential as a dietary supplement for enhancing cognition and alleviating sleep disturbances in children, providing a complementary approach alongside conventional strategies.

## Data Availability

The raw data supporting the conclusions of this article will be made available by the authors, without undue reservation.

## Footnotes

1
http://www.medcalc.org

## Glossary

ARE: Ashwagandha Root Extract
ANZCTR: Australian and New Zealand Clinical Trials Registry
BMI: Body Mass Index
BRIEF-2: Behavior Rating Inventory of Executive Function, 2nd Edition – Parent Version
BRI: Behavioral Regulation Index
COMPASS: Computerized Mental Performance Assessment System
CRI: Cognitive Regulation Index
CTRI: Clinical Trials Registry – India
DBP: Diastolic Blood Pressure
DIMS: Difficulty in Initiating and Maintaining Sleep
DOES: Disorders of Excessive Somnolence
DoA: Disorders of Arousal
ERI: Emotion Regulation Index
GCP: Good Clinical Practice
GEC: Global Executive Composition
HPA: Hypothalamic–Pituitary–Adrenal (Axis)
HPLC: High-Performance Liquid Chromatography
PROMIS-Fatigue: Patient-Reported Outcomes Measurement Information System – Fatigue Scale
SBP: Systolic Blood Pressure
SDQ: Strengths and Difficulties Questionnaire
SDSC: Sleep Disturbance Scale for Children
SH: Sleep Hyperhidrosis
SBD: Sleep Breathing Disorders
SWTD: Sleep–Wake Transition Disorders
WHO: World Health Organization
